# Cabozantinib-induced osteoblast secretome promotes survival and migration of metastatic prostate cancer cells in bone

**DOI:** 10.18632/oncotarget.20489

**Published:** 2017-08-24

**Authors:** Kai-Jie Yu, Jeffrey K. Li, Yu-Chen Lee, Guoyu Yu, Song-Chang Lin, Tianhong Pan, Robert L. Satcher, Mark A. Titus, Li-Yuan Yu-Lee, Wen Hui Weng, Gary E. Gallick, Sue-Hwa Lin

**Affiliations:** ^1^ Department of Translational Molecular Pathology, The University of Texas M. D. Anderson Cancer Center, Houston, Texas, USA; ^2^ Department of Genitourinary Medical Oncology, The University of Texas M. D. Anderson Cancer Center, Houston, Texas, USA; ^3^ The University of Texas Graduate School of Biomedical Sciences at Houston, Houston, Texas, USA; ^4^ Department of Medicine, Baylor College of Medicine, Houston, Texas, USA; ^5^ Division of Urology, Department of Surgery, Chang Gung Memorial Hospital at Linkou, Chang Gung University College of Medicine, Taoyuan, Taiwan; ^6^ Department of Chemical Engineering and Biotechnology and Graduate Institute of Biochemical and Biomedical Engineering, National Taipei University of Technology, Taipei, Taiwan; ^7^ Department of Orthopedic Oncology, University of Texas MD Anderson Cancer Center, Houston, Texas, USA

**Keywords:** cabozantinib, osteoblast, secretome, anchorage-independent growth, migration

## Abstract

Therapies that target cancer cells may have unexpected effects on the tumor microenvironment that affects therapy outcomes or render therapy resistance. Prostate cancer (PCa) bone metastasis is uniquely associated with osteoblastic bone lesions and treatment with cabozantinib, a VEGFR-2 and MET inhibitor, leads to a reduction in number and/or intensity of lesions on bone scans. However, resistance to cabozantinib therapy inevitably occurs. We examined the effect of cabozantinib on osteoblast differentiation and secretion in the context of therapy resistance. We showed that primary mouse osteoblasts express VEGFR2 and MET and cabozantinib treatment decreased osteoblast proliferation but enhanced their differentiation. A genome-wide analysis of transcriptional responses of osteoblasts to cabozantinib identified a set of genes accounting for inhibition of proliferation and stimulation of differentiation, and a spectrum of secreted proteins induced by cabozantinib, including pappalysin, IGFBP2, WNT 16, and DKK1. We determined that these proteins were upregulated in the conditioned medium of cabozantinib-treated osteoblasts (CBZ-CM) compared to control CM. Treatment of C4-2B4 or PC3-mm2 PCa cells with CBZ-CM increased the anchorage-independent growth and migration of these PCa cells compared to cells treated with control CM. These results suggest that the effect of cabozantinib on the tumor microenvironment may increase tumor cell survival and cause therapy resistance.

## INTRODUCTION

Many targeted therapies aim to modulate specific cancer signaling pathways. While these therapies are intended for targets that are expressed in cancer cells, these targets frequently are also present in stromal cells in the tumor microenvironment. The unintended targeting of the tumor stromal components may have an impact on the therapeutic efficacy or have unexpected therapy outcomes.

Cabozantinib is a tyrosine kinase inhibitor that has a high affinity for VEGFR2 and MET, with IC50 of 0.035±0.01 nM and 1.3±1.2 nM, respectively [1]. Cabozantinib was used to treat prostate cancer (PCa) bone metastasis in phase II and III clinical trials [2, 3]. Metastatic PCa cells in bone frequently induces osteoblastic bone lesions [4]. Due to the bone-forming nature of the PCa bone metastasis, bone scan is commonly used in detecting the bone lesions from PCa. One interesting observation was that cabozantinib treatments led to a reduction in the extent or intensity of bone scans in patients with PCa bone metastasis [2, 3]. While the results of phase II clinical trials of cabozantinib on PCa bone metastasis were promising, the phase III clinical trial fails to show improvement in patients’ survival [3]. However, cabozantinib was shown to improve outcomes of other cancers and has been approved for the treatment of advanced renal cell carcinoma in patients who have received prior anti-angiogenic therapy [5].

Several possible mechanisms of resistance to cabozantinib treatments in PCa have been reported. Varkaris et al. [6] have reported that large vessels that express VEGFR1 in the tumors are resistant to cabozantinib treatments. Lee et al. [7] demonstrated that PCa-induced bone provides integrin ligands that support tumor cell survival. Because integrin ligands are present in the tumor microenvironment before cabozantinib administration, this constitutes a de novo mechanism of therapy resistance. Subsequent studies in animal model by Dai et al. [8], Nguyen et al. [9], and Varkaris et al. [6] showed that cabozantinib increases osteoblast differentiation *in vitro* and bone formation *in vivo*, suggesting cabozantinib has a direct effect on osteoblasts. Whether the effect of cabozantinib on osteoblasts promotes therapy resistance to cabozantinib has not been examined.

In this study, we used gene expression profile analysis to identify cellular pathways that are modulated by cabozantinib. These alterations are a result of MET or VEGFR2 inhibition in osteoblasts. In addition, we found that cabozantinib induces the secretion of a spectrum of proteins from osteoblasts. These secreted proteins could act as paracrine factors to provide therapy resistance, suggesting that the unintended targeting of the tumor stromal components by cabozantinib may have an impact on the therapy outcomes.

## RESULTS

### Expression of MET and VEGFR2 in osteoblasts

**Figure 1:**
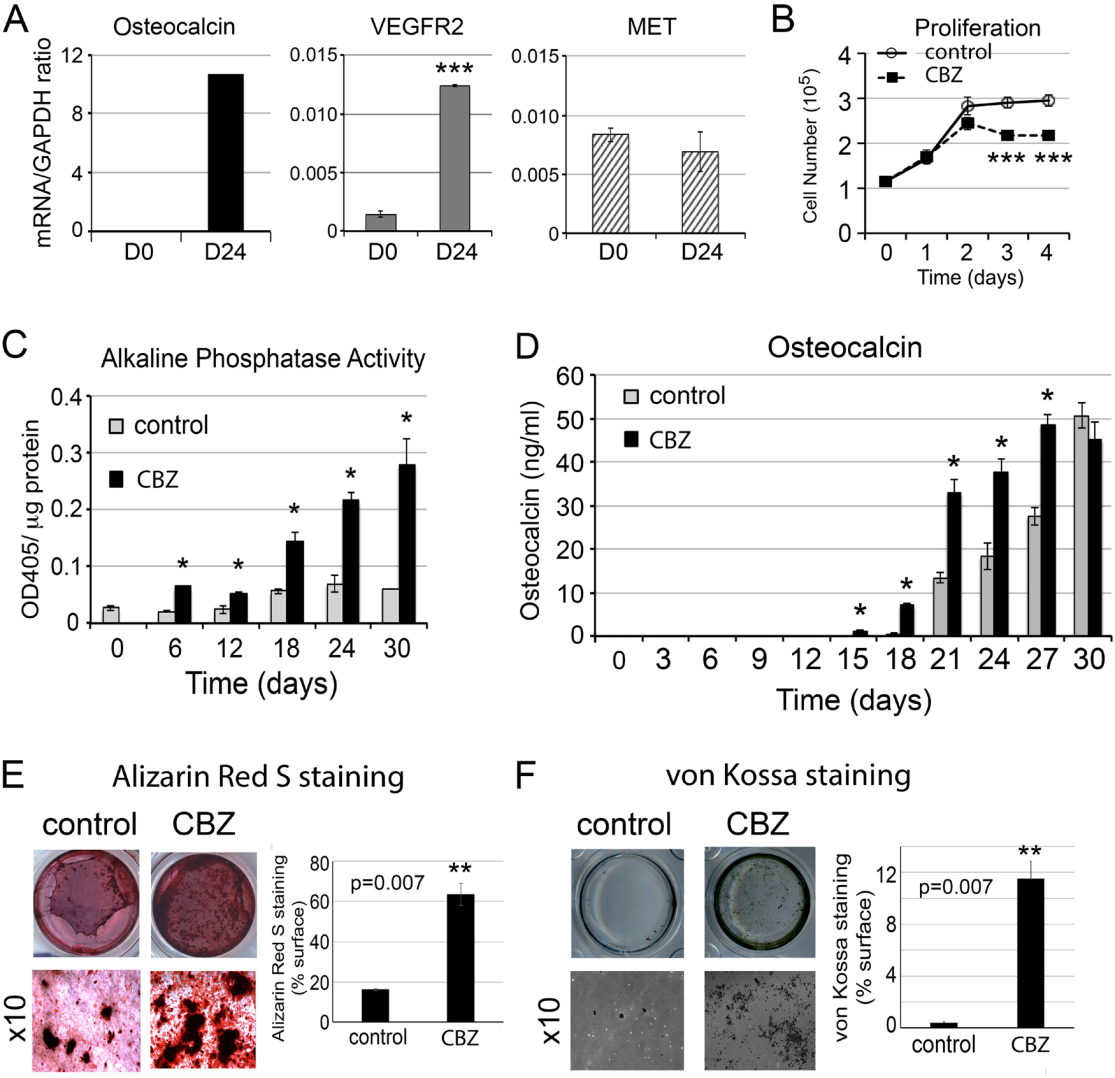
Effect of cabozantinib on osteoblast proliferation and differentiation **(A)** Real-time RT-PCR for the expression of osteocalcin, VEGFR2, and MET in the undifferentiated (D0) and differentiated (D24) osteoblasts; **(B)** Effects of cabozantinib on osteoblast proliferation measured by cell counting; **(C)** Effects of cabozantinib on osteoblast alkaline phosphatase activity during the time course of osteoblast differentiation; **(D)** Effects of cabozantinib on osteocalcin protein secretion measured by ELISA; and **(E)** Effects of cabozantinib on osteoblast mineralization measured by Alizarin Red S staining and **(F)** von Kossa staining. *, p<0.05, **, p<0.01, ***, p<0.001.

To examine whether osteoblasts could be a target of cabozantinib, we examined whether MET and VEGFR2 are expressed in osteoblasts. Primary mouse osteoblasts (PMOs) were isolated from newborn mouse calvaria and cultured to confluence (D0 osteoblasts). To prepare differentiated PMOs, the D0 osteoblasts were further cultured in differentiation medium for 24 days (D24 osteoblasts), which resulted in an increase in the expression of osteocalcin, a marker of osteoblast differentiation, compared to D0 osteoblasts (Figure 1A, left panel). The expression of VEGFR2 and MET in D0 and D24 osteoblasts were examined by real-time RT-PCR. The oligonucleotide primers used in this study is shown in Supplementary Table 1. Real-time PCR showed that D0 osteoblasts express a low amount of VEGFR2, as indicated by the VEGFR2/GAPDH ratio (Figure 1A, middle panel). However, the levels of VEGFR2 are increased 8.5-fold in D24 osteoblasts compared to D0 osteoblasts (Figure 1A, middle panel). These observations are consistent with those reported by Deckers et al. [10], which showed that mouse preosteoblast-like cell line KS483 expressed VEGFR2 during mineralization. Real-time RT-PCR showed that MET is also expressed in PMOs, but the levels of MET in D0 and D24 osteoblasts are similar (Figure 1A, right panel). These results indicate that both MET and VEGFR2 are expressed in primary mouse osteoblasts.

### Cabozantinib inhibits proliferation but enhances differentiation of osteoblasts

We examined the effects of cabozantinib on the proliferation of PMOs. As shown in Figure 1B, cabozantinib inhibited the proliferation of calvarial osteoblasts at a concentration of 100 nM. To examine the effect of cabozantinib on osteoblast differentiation, confluent osteoblasts were cultured in differentiation medium containing β-glycerol phosphate and ascorbic acid for 6, 18, 24, and 30 days with or without cabozantinib. Cabozantinib treatment led to a significant increase in the alkaline phosphatase activity, a marker of osteoblast differentiation, throughout the entire time course of differentiation (Figure 1C). Cabozantinib also increases the levels of osteocalcin in the conditioned medium up to 27 days as measured by ELISA (Figure 1D), and the mineralization of osteoblasts as measured by alizarin red S (Figure 1E) and von Kossa staining (Figure 1F). These observations indicate that cabozantinib treatment increases osteoblast differentiation.

### Transcriptional responses of osteoblasts to cabozantinib treatments

**Figure 2:**
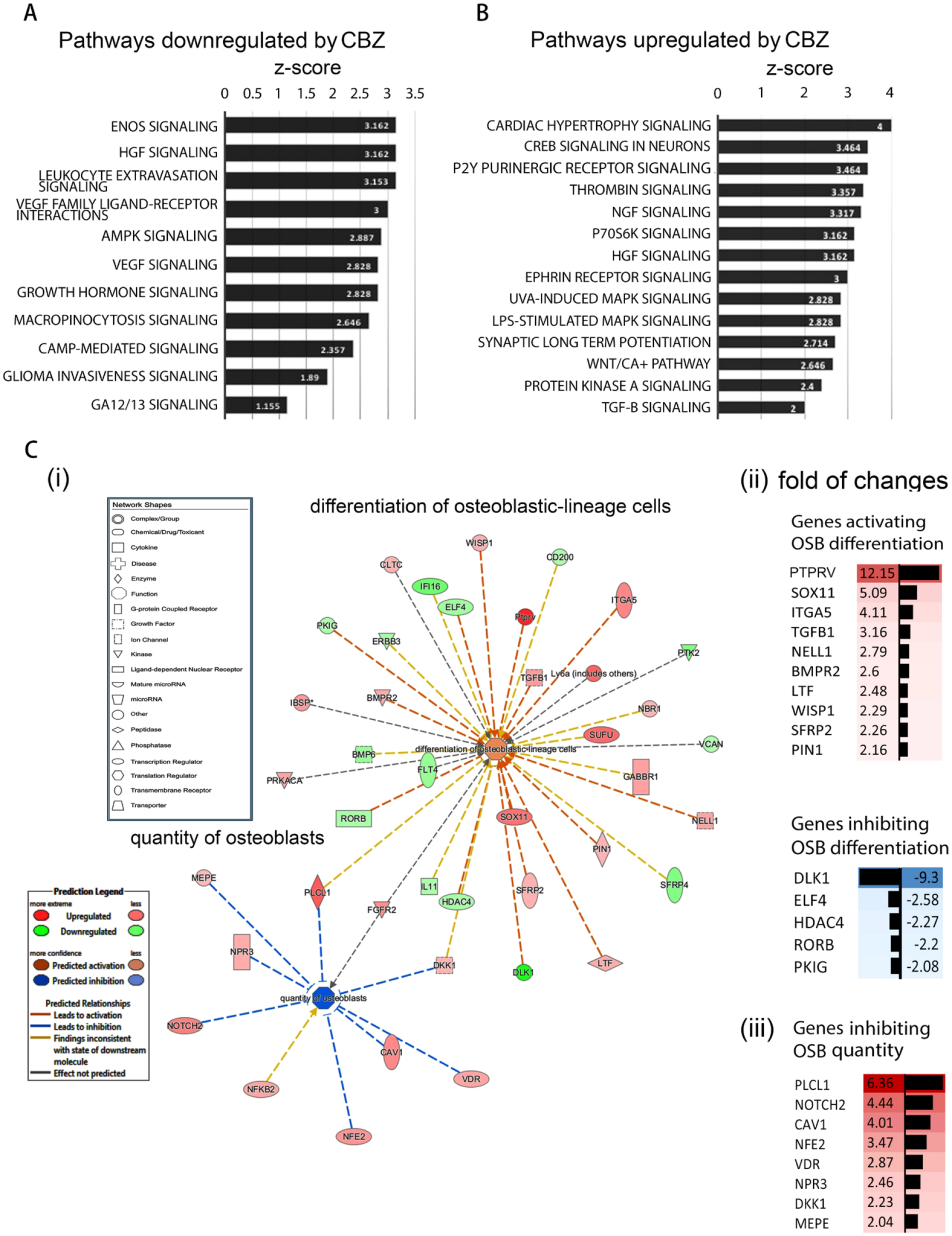
Pathways affected by cabozantinib treatments using ingenuity pathway analysis (IPA) **(A)** Pathways that may be affected by genes up-regulated by cabozantinib; **(B)** Pathways that may be affected by genes down-regulated by cabozantinib; **(C)** (i) Cabozantinib regulated genes that are involved in “differentiation of osteoblastic-lineage cells” and “inhibition of osteoblast proliferation” based on IPA; (ii) fold of change of up-regulated genes and down-regulated genes related to inhibition of osteoblast differentiation. (iii) fold of change of up-regulated genes related to inhibition of osteoblast quantity.

It is likely that cabozantinib enhances osteoblast differentiation through inhibition of VEGFR2 and MET-mediated signaling pathways, and this inhibition leads to changes in gene expression. To examine the effects of cabozantinib on the gene expression of osteoblasts, gene array profiling of D24 osteoblasts with or without cabozantinib treatment was performed. Genes that were upregulated or downregulated more than 2 fold and have a p-value < 0.05 were considered for further analysis. The resulting 2944 genes were analyzed for the pathways involved. In silico analysis with Ingenuity Pathway Analysis (IPA) was used to detect biological pathways altered by cabozantinib treatment. As shown in Figure 2A, pathways involving HGF (a MET ligand) signaling, VEGF family ligand receptor interaction, and VEGF signaling pathways were found to be downregulated by cabozantinib, consistent with cabozantinib inhibition of MET and VEGFR2. In addition to these known targets, we also found that eNOS signaling, leukocyte extravasation signaling, AMPK signaling, and growth hormone signaling pathways in osteoblasts are downregulated by cabozantinib (Figure 2A). Pathways that are upregulated by cabozantinib were also observed. These include cardiac hypertrophy signaling, CREB signaling in neurons, P2Y purinergic receptor signaling, and NGF signaling (Figure 2B).

### Genes that enhance osteoblast differentiation

Next, we used IPA to analyze genes whose changes may be relevant to osteoblast activities. IPA analyses identified 20 genes whose upregulation and 14 genes whose downregulation are associated with “differentiation of osteoblastic-lineage cells” (Figure 2Ci, Table 1, and Table 2). Thus, the analyses predicted that cabozantinib treatment may lead to an increase of osteoblast differentiation (Figure 2Ci). PTPRV, SOX11, ITGA5, TGFB1, NELL1, BMPR2, LTF, WISP1, SFRP2, and PIN1, are among the upregulated genes that are associated with osteoblast differentiation (Figure 2Ci and Figure 2Cii). PTPRV, protein tyrosine phosphatase receptor type V, also known as osteotesticular protein tyrosine phosphatase (OST-PTP) [11], was upregulated 12-fold by cabozantinib treatment in gene array analysis (Figure 2Cii). PTPRV was previously shown to increase osteoblast differentiation [12] and SOX 11 and NELL1 has been shown to play a role in skeletal ossification [13].

**Table 1:** Cabozantinib upregulated genes that are involved in the “differentiation of osteoblastic-lineage cells”

Symbol	Gene Name	Fold Change	p-value	Location	Family
PTPRV	protein tyrosine phosphatase, receptor type, V	12.15	0.022	*Other	**other
SOX11	SRY-box 11	5.09	0.001	Nucleus	transcription regulator
ITGA5	integrin subunit alpha 5	4.11	0.005	Plasma Membrane	transmembrane receptor
TGFB1	transforming growth factor beta 1	3.16	0.001	Extracellular Space	growth factor
NELL1	neural EGFL like 1	2.79	1.00E-03	Extracellular Space	growth factor
BMPR2	bone morphogenetic protein receptor type 2	2.6	0.006	Plasma Membrane	kinase
LTF	lactotransferrin	2.48	0.031	Extracellular Space	peptidase
WISP1	WNT1 inducible signaling pathway protein 1	2.29	0.003	Extracellular Space	other
SFRP2	secreted frizzled-related protein 2	2.26	0.016	Plasma Membrane	transmembrane receptor
PIN1	peptidylprolyl cis/trans isomerase, NIMA-interacting 1	2.16	0.039	Nucleus	enzyme
PLCL1	phospholipase C like 1	6.36	0.0065	Cytoplasm	enzyme
SUFU	SUFU negative regulator of hedgehog signaling	5.61	0.004	Nucleus	transcription regulator
GABBR1	gamma-aminobutyric acid type B receptor subunit 1	2.96	4.00E-04	Plasma Membrane	G-protein coupled receptor
DKK1	dickkopf WNT signaling pathway inhibitor 1	2.23	0.047	Extracellular Space	growth factor
NBR1	neighbor of BRCA1 gene 1	2.15	0.041	Cytoplasm	other
Ly6a (includes others)	lymphocyte antigen 6 complex, locus A	5.94	0.014	Plasma Membrane	other
FGFR2	fibroblast growth factor receptor 2	3.71	0.026	Plasma Membrane	kinase
IBSP	integrin binding sialoprotein	3.22	1.64E-05	Extracellular Space	other
PRKACA	protein kinase cAMP-activated catalytic subunit alpha	2.9	0.003	Cytoplasm	kinase
CLTC	clathrin heavy chain	2.25	0.048	Plasma Membrane	other

* The location of the protein has not been defined.

** The function of the protein has not been assigned.

**Table 2:** Cabozantinib downregulated genes that are involved in the “differentiation of osteoblastic-lineage cells”

Symbol	Gene Name	Fold Change	p-value	Location	Family
DLK1	delta-like 1 homolog (Drosophila)	-9.30	0.010	Extracellular Space	**other
ELF4	E74 like ETS transcription factor 4	-2.58	0.003	Nucleus	transcription regulator
HDAC4	histone deacetylase 4	-2.27	0.022	Nucleus	transcription regulator
RORB	RAR related orphan receptor B	-2.20	0.016	Nucleus	ligand-dependent nuclear receptor
PKIG	protein kinase (cAMP-dependent, catalytic) inhibitor gamma	-2.08	0.018	*Other	other
IL11	interleukin 11	-2.12	0.007	Extracellular Space	cytokine
CD200	CD200 molecule	-2.23	0.002	Plasma Membrane	other
ERBB3	erb-b2 receptor tyrosine kinase 3	-2.29	0.018	Plasma Membrane	kinase
BMP6	bone morphogenetic protein 6	-2.79	0.042	Extracellular Space	growth factor
SFRP4	secreted frizzled related protein 4	-3.56	0.004	Plasma Membrane	transmembrane receptor
IFI16	interferon gamma inducible protein 16	-4.48	0.018	Nucleus	transcription regulator
VCAN	versican	-2.01	0.023	Extracellular Space	other
FLT4	fms related tyrosine kinase 4	-2.83	0.035	Plasma Membrane	transmembrane receptor
PTK2	protein tyrosine kinase 2	-3.62	0.008	Cytoplasm	kinase

* The location of the protein has not been defined.

** The function of the protein has not been assigned.

**Figure 3:**
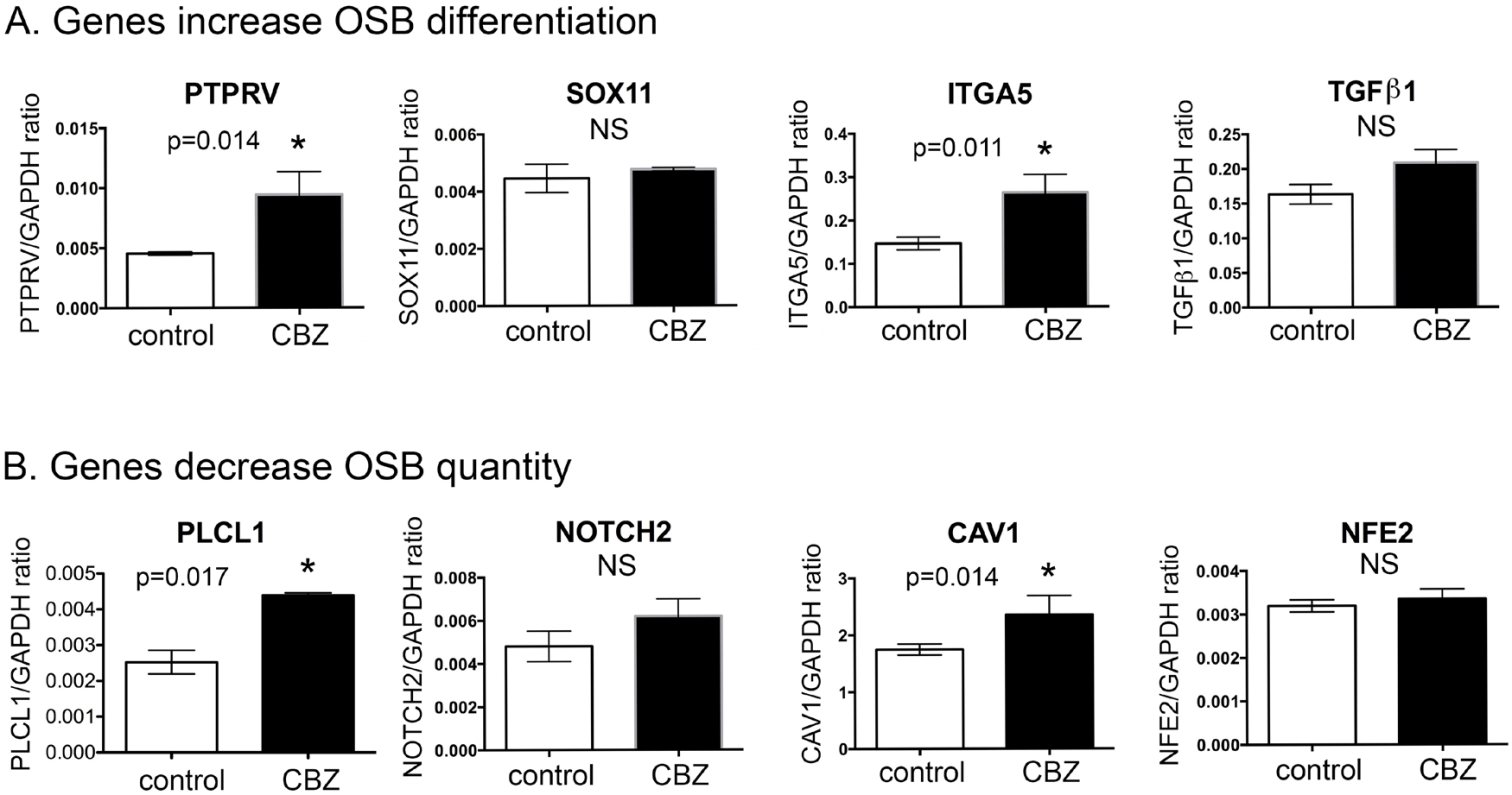
Real-time RT-PCR for mRNA levels of cabozantinib regulated genes involved in “differentiation of osteoblastic-lineage cells” and “inhibition of osteoblast proliferation” **(A)** Real-time RT-PCR for the mRNAs of genes involved in increase of osteoblast differentiation; **(B)** Real-time RT-PCR for the mRNAs of genes involved in the inhibition of osteoblast proliferation. *, p<0.05.

To verify the gene array analysis, we tested genes that showed more than a 3-fold increase, i.e., PTPRV, SOX11, ITGA5 and TGFβ1, by real-time RT-PCR. For each specific gene examined, three pairs of primers were tested for their specificities and the best oligo pair, which generated high level of predicted product without non-specific products, was selected for real-time RT-PCR analysis (data not shown). Real-time RT-PCR of the messages prepared from D24 osteoblasts treated with or without cabozantinib showed that the mRNAs of PTPRV and ITGA5 are upregulated by cabozantinib, while SOX11 and TGFβ1 do not show significant changes in the levels of mRNAs by cabozantinib (Figure 3A).

Among the downregulated genes, DLK1 was downregulated >9-fold by cabozantinib treatment (Figure 2Cii). DLK1 was reported to be associated with inhibition of osteoblast differentiation [14]. However, the DLK1 mRNA levels in D24 osteoblasts with or without cabozantinib treatment were too low to be accurately determined (data not shown).

### Genes that inhibit osteoblast proliferation

Next, we analyzed the genes whose changes may be relevant to osteoblast proliferation. IPA analyses identified 10 genes whose upregulation is associated with a decrease of “quantity of osteoblasts” (Figure 2Ci and Table 3). Thus, the analyses predicted that cabozantinib treatment might lead to inhibition of osteoblast quantity (Figure 2Ci). PLCL1, NOTCH2, CAV1, NFE2, VDR, NPR3, DKK1, and MEPE are among the upregulated genes (Table 3, Figure 2Ciii). Four genes attained the criteria of >3-fold change and real-time RT-PCR was performed to determine mRNAs levels in D24 osteoblasts treated with or without cabozantinib. As shown in Figure 3B, the mRNAs of PLCL1 and CAV1 are upregulated by cabozantinib while those of NOTCH2 and NFE2 are not (Figure 3B). These observations suggest that upregulation of these genes by cabozantinib inhibits osteoblast proliferation.

**Table 3:** Cabozantinib regulated genes that are involved in “quantity of osteoblasts”

Symbol	Gene name	Fold Change	p-value	Location	Family
MEPE	matrix extracellular phosphoglycoprotein	2.04	0.003	Extracellular Space	**other
PLCL1	phospholipase C like 1	6.36	0.006	Cytoplasm	enzyme
VDR	vitamin D (1, 25- dihydroxyvitamin D3) receptor	2.87	0.007	Nucleus	transcription regulator
NPR3	natriuretic peptide receptor 3	2.46	0.014	Plasma Membrane	G-protein coupled receptor
NFE2	nuclear factor, erythroid 2	3.47	0.015	Nucleus	transcription regulator
CAV1	caveolin 1	4.01	0.030	Plasma Membrane	transmembrane receptor
NOTCH2	notch 2	4.44	0.037	Plasma Membrane	transcription regulator
DKK1	dickkopf WNT signaling pathway inhibitor 1	2.23	0.047	Extracellular Space	growth factor
NFKB2	nuclear factor kappa B subunit 2	2.74	0.043	Nucleus	transcription regulator
FGFR2	fibroblast growth factor receptor 2	3.71	0.026	Plasma Membrane	kinase

** The function of the protein has not been assigned.

### Cabozantinib induces secretion of factors from osteoblasts

**Figure 4:**
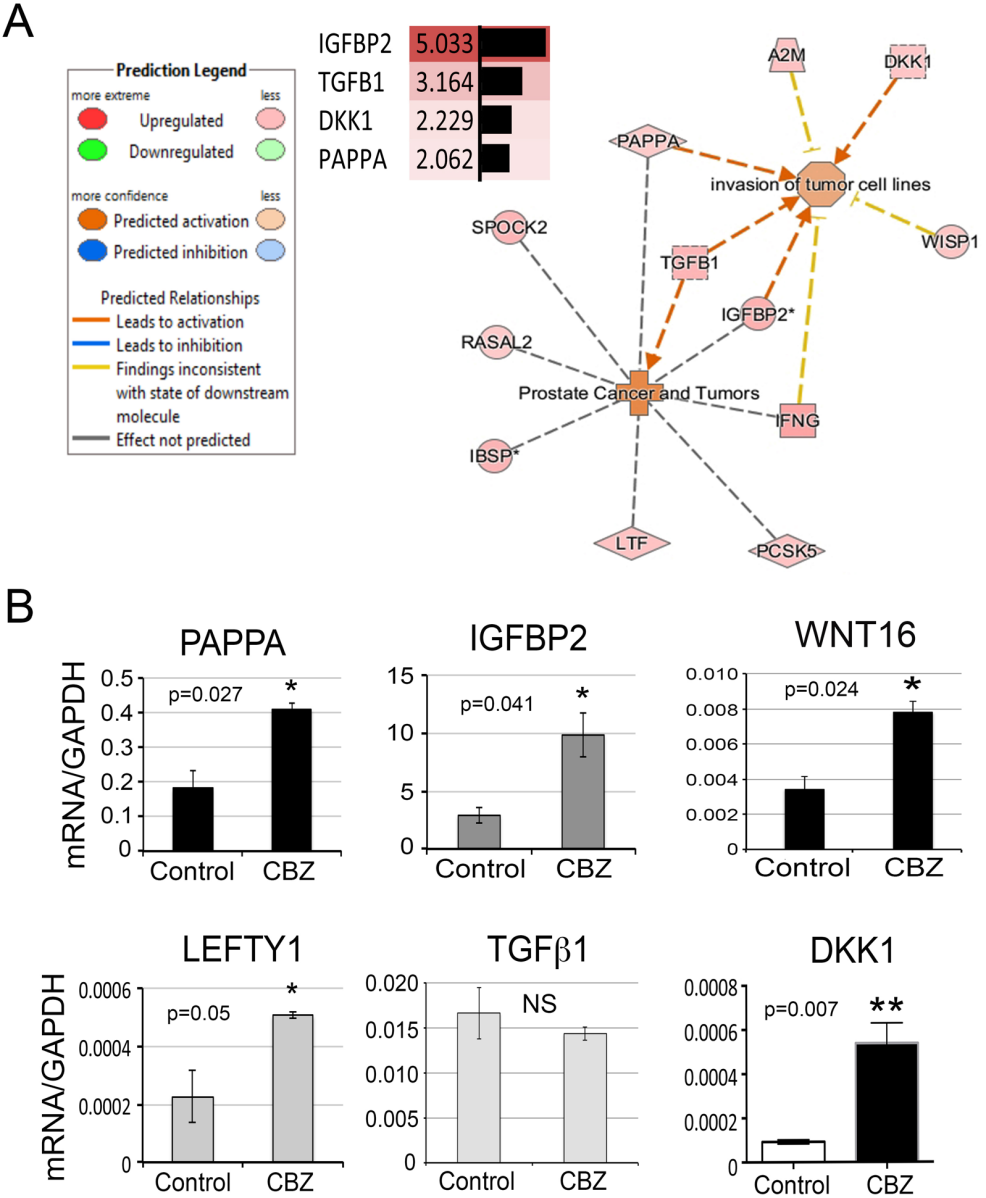
Secretory proteins upregulated by cabozantinib treatment **(A)** Secretory proteins that may be involved in “prostate cancer” and “invasion of tumor cells” from IPA. **(B)** Real-time RT-PCR for the mRNA levels of several secretory proteins in the RNAs prepared from D24 osteoblasts with or without cabozantinib treatment. *, p<0.05, **, p<0.01.

Cabozantinib treatment may affect factors secreted from osteoblasts. Because of the intimate relationship between osteoblasts and PCa cells in the bone microenvironment, alterations in osteoblast-secreted factors following cabozantinib treatment may have an effect on PCa cells through paracrine effects. Analysis of gene array results revealed 63 secreted factors that are upregulated more than 2-fold following cabozantinib treatment (Table 4). In silico analysis to map the function of these secreted factors identified PAPPA, IGFBP2, DKK1, and TGFβ1, which have documented effects on “invasion of tumor cells” or “prostate cancer and tumors” (Figure 4A). In addition, WNT16, previously shown to play a role in therapy resistance of PCa to radiation therapy [15], and LEFTY1, a TGF-β family protein required for left-right axis determination [16, 17], are also upregulated by cabozantinib (Table 4). Real-time RT-PCR showed that the mRNAs of PAPPA, IGFBP2, WNT16, LEFTY1, and DKK1 are upregulated by cabozantinib while that of TGFβ1 is not (Figure 4B).

**Table 4:** Cabozantinib upregulated genes that encode secreted factors

Symbol	Gene Name	Fold Change	p-value	Family
NELL1	neural EGFL like 1	2.794	9.25E-04	growth factor
TGFB1	transforming growth factor beta 1	3.164	1.11E-03	growth factor
NTF4	neurotrophin 4	2.059	2.75E-02	growth factor
LEFTY1	left-right determination factor 1	3.53	4.22E-02	growth factor
DKK1	dickkopf WNT signaling pathway inhibitor 1	2.229	4.71E-02	growth factor
IFNG	interferon, gamma	4.285	4.44E-03	cytokine
Ccl27a	chemokine (C-C motif) ligand 27A	3.589	1.31E-02	cytokine
IBSP	integrin binding sialoprotein	3.218	1.64E-05	**other
FAM3A	family with sequence similarity 3 member A	2.882	6.53E-04	other
WISP1	WNT1 inducible signaling pathway protein 1	2.285	2.88E-03	other
FAM132B	family with sequence similarity 132 member B	3.75	2.93E-03	other
WFDC1	WAP four-disulfide core domain 1	2.395	2.99E-03	other
MEPE	matrix extracellular phosphoglycoprotein	2.035	3.23E-03	other
CFC1/CFC1B	cripto, FRL-1, cryptic family 1	6.786	4.16E-03	other
AIM1L	absent in melanoma 1-like	2.13	4.82E-03	other
RASAL2	RAS protein activator like 2	2.119	4.96E-03	other
VMO1	vitelline membrane outer layer 1 homolog (chicken)	3.936	6.43E-03	other
FBLN1	fibulin 1	2.316	7.85E-03	other
ITIH4	inter-alpha-trypsin inhibitor heavy chain family member 4	2.663	8.95E-03	other
Hamp/Hamp2	hepcidin antimicrobial peptide	5.98	1.18E-02	other
SMTN	smoothelin	3.985	1.29E-02	other
BPIFB2	BPI fold containing family B member 2	2.534	1.62E-02	other
IGFBP2	insulin like growth factor binding protein 2	5.033	1.91E-02	other
PCOLCE2	procollagen C-endopeptidase enhancer 2	2.148	1.98E-02	other
Fcna	ficolin A	2.125	2.05E-02	other
MFAP4	microfibrillar associated protein 4	2.188	2.06E-02	other
OLFML3	olfactomedin like 3	3.071	2.36E-02	other
KLHL5	kelch like family member 5	3.402	2.49E-02	other
CRB2	crumbs family member 2	2.055	2.57E-02	other
IFT22	intraflagellar transport 22	2.547	2.73E-02	other
VSTM2A	V-set and transmembrane domain containing 2A	2.168	2.84E-02	other
IL34	interleukin 34	2.859	2.95E-02	other
IFT172	intraflagellar transport 172	5.1	2.99E-02	other
WNT16	wingless-type MMTV integration site family member 16	3.806	3.14E-02	other
ADAMTSL1	ADAMTS like 1	4.286	3.24E-02	other
SPOCK2	sparc/osteonectin, cwcv and kazal-like domains proteoglycan (testican) 2	3.193	3.39E-02	other
APCS	amyloid P component, serum	3.698	3.47E-02	other
SLIT3	slit guidance ligand 3	2.855	3.55E-02	other
COL11A2	collagen, type XI, alpha 2	4.198	3.68E-02	other
PYY	peptide YY	3.351	4.03E-02	other
WFIKKN2	WAP, follistatin/kazal, immunoglobulin, kunitz and netrin domain containing 2	2.424	4.27E-02	other
KDM6B	lysine (K)-specific demethylase 6B	2.073	4.30E-02	other
SCUBE2	signal peptide, CUB domain, EGF-like 2	2.188	4.32E-02	other
SERPINE1	serpin peptidase inhibitor	2.171	4.61E-02	other
COL22A1	collagen, type XXII, alpha 1	2.628	4.93E-02	other
LIPI	lipase, member I	9.486	4.79E-04	enzyme
GFOD2	glucose-fructose oxidoreductase domain containing 2	2.93	6.91E-04	enzyme
FUCA2	fucosidase, alpha-L- 2, plasma	2.088	3.22E-02	enzyme
LOXL1	lysyl oxidase like 1	2.721	4.61E-02	enzyme
PAPPA2	pappalysin 2	12.557	4.50E-04	peptidase
MMP8	matrix metallopeptidase 8	3.24	5.39E-04	peptidase
PAPPA	pregnancy-associated plasma protein A, pappalysin 1	2.062	1.81E-03	peptidase
CPXM2	carboxypeptidase X (M14 family), member 2	2.846	6.45E-03	peptidase
CPB1	carboxypeptidase B1	2.991	1.32E-02	peptidase
CPA3	carboxypeptidase A3	3.977	1.47E-02	peptidase
CPB2	carboxypeptidase B2	2.243	2.09E-02	peptidase
CPA4	carboxypeptidase A4	3.1	2.36E-02	peptidase
ADAMTS8	ADAM metallopeptidase with thrombospondin type 1 motif 8	2.221	2.59E-02	peptidase
Prss32	protease, serine 32	4.528	2.69E-02	peptidase
LTF	lactotransferrin	2.479	3.14E-02	peptidase
PCSK5	proprotein convertase subtilisin/kexin type 5	2.491	4.84E-02	peptidase
BPGM	bisphosphoglycerate mutase	2.414	3.26E-02	phosphatase
A2M	alpha-2-macroglobulin	2.54	3.52E-02	transporter

** The function of the protein has not been assigned.

**Figure 5:**
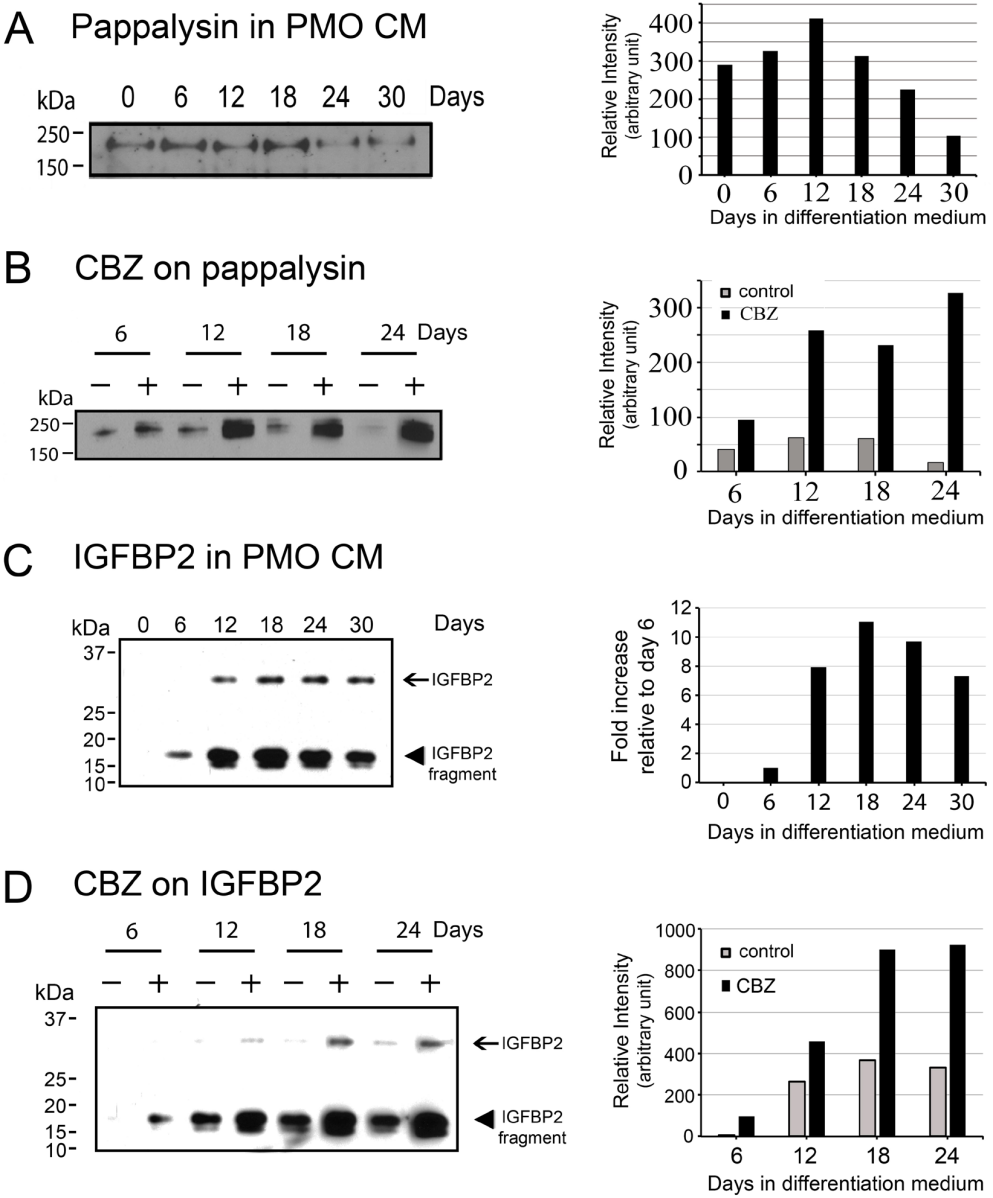
Effects of cabozantinib on PAPPA and IGFBP2 protein expression **(A)** Left panel, Western blots for PAPPA in the conditioned medium from osteoblasts at various time points of differentiation. Right panel, quantification of the intensity of PAPPA in the Western blots. **(B)** Left panel, Western blots for PAPPA in the conditioned medium of osteoblasts treated with or without cabozantinib. Right panel, quantification of the intensity of PAPPA in the Western blots showed significant increase of PAPPA by cabozantinib treatment compared to control. **(C)** Left panel, Western blots for the expression of IGFBP2 in the conditioned medium from osteoblasts at various time points of differentiation. IGFBP2 is a protein with an apparent molecular mass of around 30 kDa and it could be proteolyzed to 17 and 12 kDa fragments. Right panel, quantification of the intensity of total IGFBP2 in the Western blots showed IGFBP2 expression was increased after differentiation for 12 days and remained increased throughout the time course of differentiation. **(D)** Left panel, Western blots for IGFBP2 in the conditioned medium of osteoblasts treated with or without cabozantinib. Right panel, quantification of the intensity of total IGFBP2 in the Western blots showed significant increase of IGFBP2 by cabozantinib treatment compared to control.

Western blots were used to further confirm the expression of PAPPA and IGFBP2 in the conditioned medium from osteoblasts treated with or without cabozantinib. Western blot showed that PAPPA, a ∼170 kDa protein, is expressed at low levels in the PMO conditioned medium throughout the time course of differentiation (Figure 5A). The levels of PAPPA protein in the PMO conditioned medium were significantly increased, about 4-fold, after treating with cabozantinib for 12-24 days (Figure 5B), consistent with the changes seen in message levels (Figure 4B).

IGFBP2 is a protein with an apparent molecular weight of around 30 kDa. Western blot of the PMO conditioned media showed that IGFBP2 was proteolyzed to 17 and 12 kDa fragments (Figure 5C). Interestingly, previous studies by Gerard et al. [18] showed that IGFBP2 is proteolyzed by PAPPA in bovine and porcine growing follicles, and they hypothesized that proteolytic cleavage of IGFBP2 contributed to the increase in IGF bioavailability. Quantification of the levels of IGFBP2 by combining the intensities of the intact and proteolyzed fragments showed that the expression of IGFBP2 was increased 8- to 10-fold during PMO differentiation compared to D6 PMO, as undifferentiated D0 PMO did not have detectable levels of IGFBP2 (Figure 5C). Treatment of PMO with cabozantinib during osteoblast differentiation led to a 2- to 3-fold increase of IGFBP-2 (Figure 5D).

**Figure 6:**
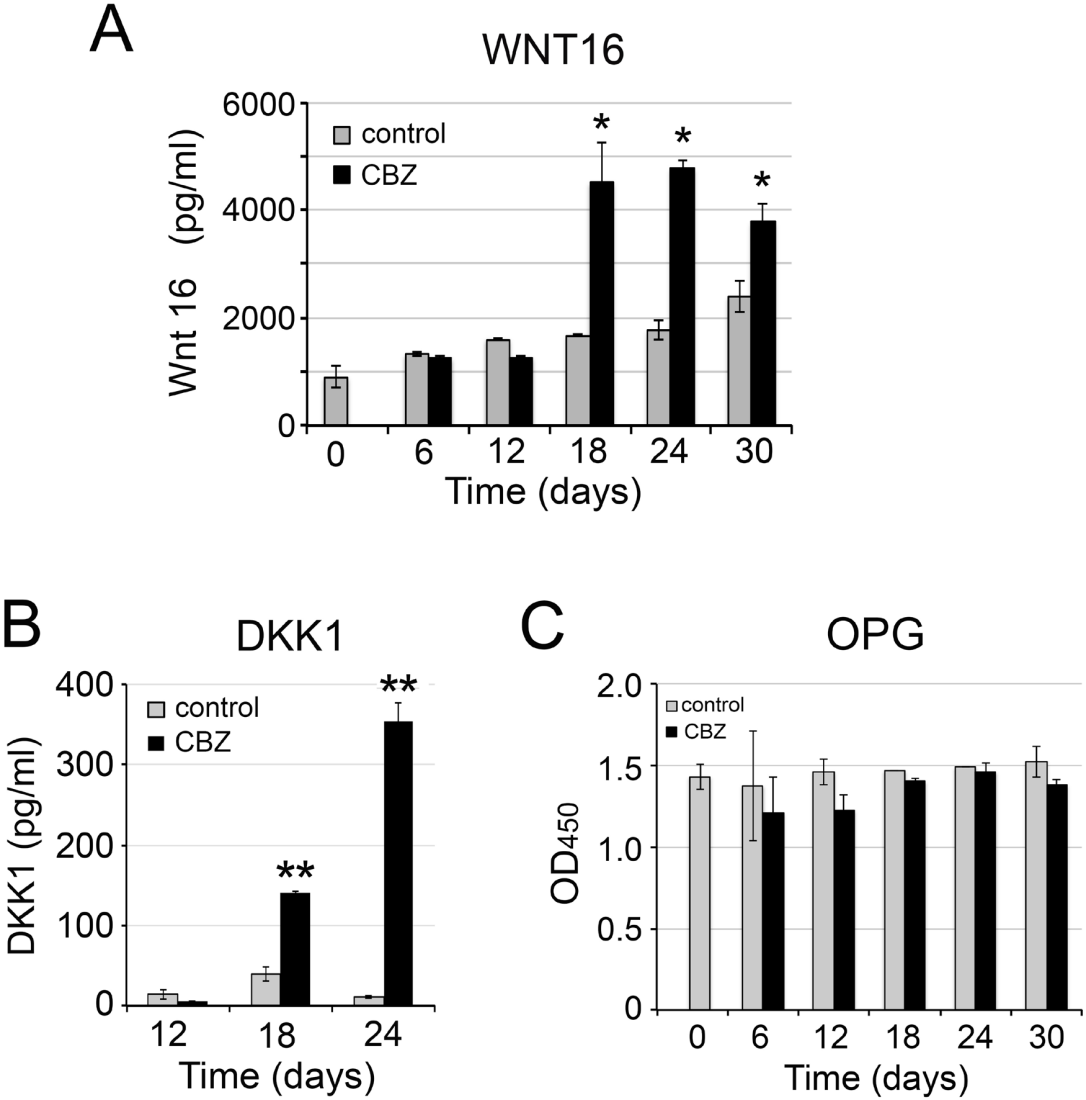
Effect of cabozantinib on WNT16, DKK1, and OPG expression ELISA of **(A)** WNT 16, **(B)** DKK1, **(C)** OPG in the conditioned medium from osteoblasts with or without cabozantinib treatments. *, p<0.05, **, p<0.01.

ELISAs were used to confirm the expression of WNT16 and DKK1 in the conditioned medium from osteoblasts treated with or without cabozantinib. As shown in Figure 6A, the amount of WNT16 protein in the PMO conditioned medium was significantly increased, about 2- to 3-fold, after 18-24 days of cabozantinib treatment (Figure 6A). Similarly, the amount of DKK1 protein in the PMO conditioned medium was also significantly increased at 18 or 24 days of cabozantinib treatment (Figure 6B).

### Effects of cabozantinib on the expression of RANKL and OPG

RANKL and OPG are two factors secreted by osteoblasts that play a critical role in the regulation of osteoclast activity. Pantano et al. reported that cabozantinib treatment increased OPG and down-regulated RANKL expression at both message and protein levels in human primary osteoblasts [19]. Stern and Alvares [20] reported that cabozantinib decreases RANKL expression in MC3T3-E1 cells. Thus, it is of interest to examine whether cabozantinib treatment affects RANKL or OPG expression in D24 osteoblasts. In the gene array analysis, we found that the expression of RANKL and OPG in the cabozantinib-treated osteoblasts was 0.99-fold and 1.07-fold, respectively, compared to control, indicating that cabozantinib may not have an effect on RANKL or OPG expression in differentiated PMOs. Real time RT-PCR for the expression of RANKL in undifferentiated (D0) and differentiated (D24) osteoblasts showed that the mRNAs of RANKL was too low to be determined (data not shown). RANKL protein in PMO CMs with or without cabozantinib treatments was also undetectable by ELISA (data not shown). Similarly, ELISA for OPG showed that cabozantinib treatment did not affect OPG protein levels during osteoblast differentiation (Figure 6C). These observations are consistent with the gene array analysis. Together, these results suggest that cabozantinib treatment may not have significant effects on RANKL or OPG expression in mouse PMOs.

### Conditioned medium from cabozantinib-treated osteoblasts increases anchorage-independent growth and migration of PCa cells

**Figure 7:**
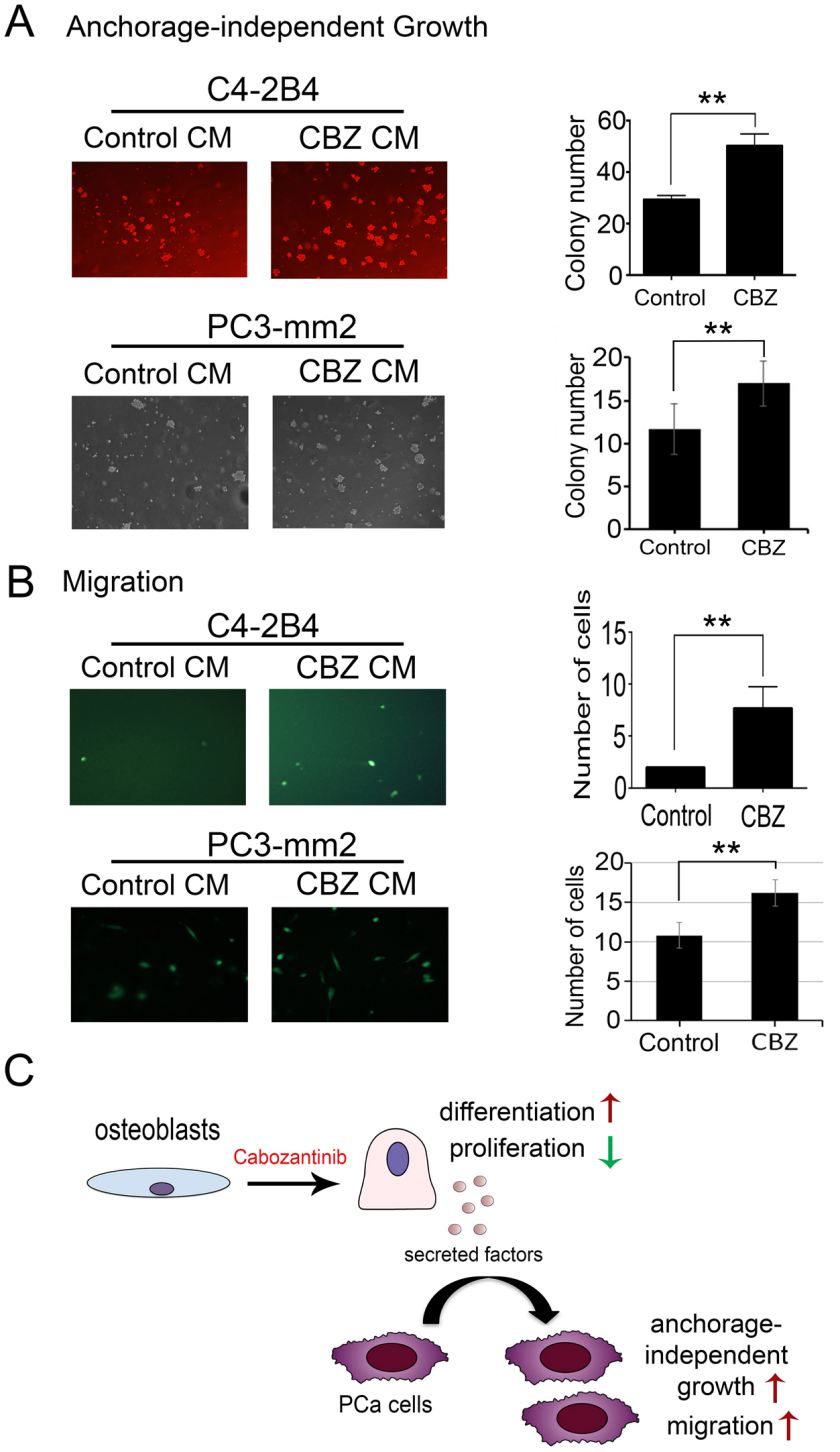
Effect of conditioned medium from cabozantinib-treated osteoblasts on anchorage-independent growth and migration of C4-2B4 or PC3-mm2 prostate cancer cells **(A)** Soft agar colony assay showed CBZ CM significantly increase C4-2B4 colonies compared to control CM. **(B)** CBZ CM increased the migration of C4-2B4 cells compared to control CM in Transwell migration assay. **(C)** Diagram illustrates effects of cabozantinib on osteoblasts and cabozantinib-induced secreted factors may promote survival and migration of PCa cells. **, p<0.01.

Cabozantinib-induced secreted factors from osteoblasts may affect the survival of the tumor cells during cabozantinib treatments. Among the cabozantinib-induced secreted factors, PAPPA was previously shown to be a stroma-secreted factor that can activate NFκB signaling in hepatocellular carcinoma (HCC) cells, and advanced stage HCC has higher expression levels of PAPPA [21]. Increased IGFBP2 was shown to stimulate the proliferation of androgen-independent PCa cells [22, 23] and its expression was shown to be associated with progression of PCa to androgen-independent state [24–26]. WNT16 was previously shown to play a role in therapy resistance [15]. Together, the cabozantinib-induced factors may promote tumor cell survival in a paracrine manner. Thus, we examined the effects of conditioned media from cabozantinib-treated (CBZ-CM) versus untreated (CM) D24 osteoblasts on the survival of PCa cells. At D24, the osteoblasts were fully mineralized (Figure 1). Equal volumes of CBZ-CM and CM were used in the studies. Treatment of C4-2B4 or PC3-mm2 PCa cells with CBZ-CM increased the anchorage-independent growth of C4-2B4 and PC3-mm2 cells compared to those treated with CM (Figure 7A), while treatment of C4-2B4 or PC3-mm2 cells with 100 nM cabozantinib alone did not have a significant effect (Supplementary Figure 1). These results suggest that cabozantinib-induced factors from osteoblasts promote tumor cell survival.

To examine whether cabozantinib-induced secreted factors can activate signaling pathways that affect cell migration, we treated PCa cells with CBZ-CM in a Transwell migration assay. C4-2B4 cells migrated at a very low speed in Transwell migration assay (Figure 7B). Treatment of C4-2B4 cells with CBZ-CM significantly increased the migration of C4-2B4 cells compared to the control CM-treated cells (Figure 7B). Similar results were observed with PC3-mm2 cells, however, to a lesser extent, likely due to the highly migratory characteristics of PC3-mm2 cells. Treatment of C4-2B4 or PC3-mm2 cells with 100 nM cabozantinib alone did not have a significant effect on cell migration (Supplementary Figure 1). Together, these results suggest that cabozantinib-induced secreted factors have effects on PCa cells and may contribute to the therapy resistance to cabozantinib.

## DISCUSSION

Our studies address an important issue on the impact of targeted therapy on the tumor microenvironment. We found that cabozantinib treatment leads osteoblasts to undergo differentiation and induces a spectrum of secreted proteins from osteoblasts. Some of these secreted proteins increase PCa cell survival and migration. Because PCa bone metastasis is frequently associated with osteoblastic bone lesions, the effects of cabozantinib on the tumor microenvironment may render therapy resistance as frequently observed in the treatment of patients with PCa bone metastasis.

Our studies suggest that stromal response to cabozantinib therapy may influence the therapy outcomes. Cabozantinib has high affinity for VEGFR2, which is mainly expressed in endothelial cells. In addition to endothelial cells, osteoblasts are prominently present in bone lesions from metastatic PCa. While endothelial cells show pronounced apoptotic response, osteoblasts respond to cabozantinib with enhanced differentiation. In addition, cabozantinib treatment leads osteoblasts to secrete factors that have effects on PCa cell survival. It is possible that cabozantinib’s effect on osteoblasts contributes to the lack of survival improvement in phase III clinical trial [3]. Because cabozantinib has been approved for the treatment of advanced renal cell carcinoma in patients who have received prior anti-angiogenic therapy, further studies on the effects of cabozantinib on tumor-associated stroma are warranted.

In osteoblasts, MET likely plays a role in supporting osteoblast proliferation. Grano et al. [27] showed that HGF, the ligand for MET, stimulates MET receptor kinase activity and MET autophosphorylation in osteoblasts. They also showed that osteoblasts respond to HGF by entering the cell cycle, as indicated by stimulation of DNA synthesis [27]. Consistently, we found that cabozantinib treatment leads to inhibition of osteoblast proliferation. Our transcriptome analysis of cabozantinib-treated osteoblasts unveils proliferation related genes whose expression is modulated by cabozantinib, providing a molecular basis for the inhibition of osteoblast proliferation. The changes in the expression of these genes are likely due to kinase inhibition, which lead to transcriptional reprogramming of osteoblasts. Other tyrosine kinase inhibitors have also demonstrated effects on osteoblasts. We have previously shown that inhibition of Src/Abl family kinase activity by dasatinib inhibited osteoblast proliferation and increased osteoblast differentiation [28]. Imantinib mesylate that inhibits several tyrosine kinases, including BCR-ABL, PDGFR alpha and beta, and the C-KIT receptor, has also been shown to inhibit osteoblast proliferation and stimulate osteoblast differentiation *in vitro* [29]. Thus, inhibition of osteoblast proliferation coupled with enhancement of osteoblast differentiation seems to be a common effect of inhibitors that target tyrosine kinases.

One of the clinical responses to cabozantinib treatment in men with PCa and bone metastasis is the reduction of the intensity of lesions on bone scans [3, 6, 30]. Because positivity in bone scan reflects new bone formation, these clinical outcomes suggest that cabozantinib decreases tumor-induced bone formation. The improvement in the intensity of lesions in bone scan from cabozantinib treatment is likely due to drug effects on inhibiting tumor angiogenesis, which reduces tumor volume and in turn results in a decrease in tumor-induced new bone formation. However, Phase III study of cabozantinib in previously treated metastatic castration-resistant PCa patients showed that cabozantinib did not have an effect on PSA outcomes [3]. Our study implies that one of the reasons for the improved bone scans is inhibition of osteoblast proliferation that leads to decreased new bone formation, providing the cause of the disconnect between bone scan improvement and clinical progression.

The cabozantinib concentration used in this *in vitro* study is 100 nM. It is possible that different concentrations of cabozantinib might result in differential transcriptomic changes in osteoblasts. However, the concentration of cabozantinib in patients’ bone is unknown. The dose of cabozantinib used in patients is 60 mg per day [3], which is around 1 mg/kg. In animal studies by Haider et al [31], the dose used was 30 mg/kg and that in Patnaik et al. [32] was 100 mg/kg. Studies by Patnaik et al. [32] measured the steady-state intratumoral concentration of cabozantinib and found it to be approximately 10 μmol/L within 72 hours. Although the concentration in the mouse bone may not be comparable to those in human, if we calculate the concentration in bone based on the dose of cabozantinib used in human versus mouse, i.e., 1 mg/kg versus 100 mg/kg, the concentration of cabozantinib in patients’ bone would be approximately 0.1 μmol/L. Thus, the cabozantinib concentration we used may be close to the cabozantinib concentration in patients’ bone.

With a significant increase in drugs available for cancer treatment, acquired resistance to anticancer treatments becomes an important issue in cancer therapy. While tumor cells have been frequently shown to acquire therapy resistance through adaptation to anticancer treatments, treatment-induced damage to the tumor microenvironment can also contribute to therapy resistance. For example, radiation-induced WNT16 expression from tumor stroma was shown to promote tumor cell survival, resulting in attenuated effects of cytotoxic chemotherapy [15]. Interestingly, we found that cabozantinib induces a spectrum of secreted proteins, including WNT16, from osteoblasts. Resistance to cabozantinib treatments has been seen in the treatment of PCa bone metastasis [3]. Three resistance mechanisms to cabozantinib therapy have been reported so far. The first mechanism is “de novo resistance”, in which the tumor-induced bone secretes factors that activate integrin and confer a survival advantage to tumor cells [7]. The second mechanism involves vascular heterogeneity. Vakaris et al. [6] showed that viable tumor cells surrounding VEGFR1-positive vessels were resistant to cabozantinib treatments. The third mechanism is that targeting MET kinase leads to the activation of FGFR1 that promotes tumor proliferation and metastatic growth in bone [6]. Our studies reveal a fourth resistance mechanism, in which cabozantinib induces the secretion of a spectrum of proteins, e.g. PAPPA and IGFBP2 that are known to increase tumorigenic properties of PCa cells, from osteoblasts to increase the survival and migration of tumor cells.

In summary, our studies indicate that targeted therapy may generate diverse responses in different cell types present in tumors. Our studies raise an important issue on the impact of tumor microenvironment and therapy outcome. Some of the identified secreted factors may be suitable as biomarkers for assessing emerging therapy resistance. Understanding the effect of cabozantinib on osteoblasts may provide rationale for combination therapies that could overcome resistance.

## MATERIALS AND METHODS

### Treatment of calvarial osteoblasts with cabozantinib

Primary mouse osteoblasts (PMO) were isolated from calvaria of 2-5 day old newborn mice. Calvariae were digested with collagenase (0.1 mg/mL) in α-MEM with trypsin (12.5 μg/mL). The first two digestions were discarded and the calvariae were further digested with 0.2 mg/mL collagenase. The osteoblasts in the supernatants and the remaining calvariae were collected and cultured in α-MEM plus 10% fetal bovine serum. Osteoblasts were collected from the culture by trypsin digestion. For proliferation assay, PMOs cultured in α-MEM plus 10% fetal bovine serum (FBS) were treated with or without 100 nM cabozantinib (Exelixis Inc. through Material Transfer Agreement). For the differentiation assay, PMOs that were grown to confluence were cultured in differentiation medium (α-MEM, 10% FBS, 100 μg/ml ascorbic acid, and 5 mM β-glycerol phosphate) with or without 100 nM cabozantinib for 30 days with a medium change every three days. The conditioned media (CMs) was collected and stored at -20°C until used. PMOs were washed with phosphate buffered saline (PBS) and either scraped from the plate in 0.5% Triton X-100/PBS solution for alkaline phosphatase activity measurement or in Trizol for RNA preparation.

### RNA preparation and real-time RT-PCR

Total RNAs were prepared from control untreated PMOs and cabozantinib-treated PMOs by using Trizol (Invitrogen). RNAs were further purified by RNeasy mini kit (Qiagen, Valencia, CA, USA) and cDNAs prepared by using TagMan Reverse Transcription Reagent kit (Applied Biosystems, Foster City, CA, USA). cDNA (20 ng) was used in quantitative real-time PCR with SYBR Green (Applied Biosystems), using *GAPDH* as a control. For each gene of interest, three mouse-specific PCR primers were selected and verified, based on the predicted sizes of PCR products and the lack of non-specific PCR products, using cDNAs from MC3T3-E1 pre-osteoblastic cell line or from 2H11 endothelial cell line. The best primer set was selected for real-time RT-PCR studies. The PCR primer sequences are listed in Supplementary Table 1.

### Proliferation assay

Primary mouse osteoblasts cultured in α-MEM medium plus 10% FBS were treated with or without 100 nM cabozantinib. Cells were trypsinzed from the culture plate at the indicated times and the cell number counted with a hemocytometer.

### Measurement of alkaline phosphatase activity

Primary mouse osteoblasts cultured in differentiation medium (α-MEM, 10% FBS, 100 μg/ml ascorbic acid, and 5 mM β-glycerol phosphate) were treated with or without 100 nM cabozantinib. Cells were lysed by using Diethanolamine Substrate Buffer (Thermo Scientific) at indicated time points. Alkaline phosphatase activity in the cell lysate was assayed by using p-nitrophenylphosphate as substrate and the absorbance measured at 405 nm.

### ELISA

Conditioned media from primary mouse osteoblasts cultured in differentiation medium with or without treating with 100 nM cabozantinib for various duration of time were collected. The levels of specific protein in conditioned medium were measured using ELISA kits from the following source: Osteocalcin (Alfa Aesar, Tewksbury, MA, USA), WNT16 (LifeSpan BioSciences, Seattle, WA, USA), DKK1 (R&D systems, Minneapolis, MN, USA), OPG (RayBiotech, Norcross, GA, USA), and RANKL (R&D systems, Minneapolis, MN, USA).

### Mineralization assay

For alizarin red S staining (Sigma, St Louis, MO, USA), primary mouse osteoblasts cultured in differentiation medium with or without treating with 100 nM cabozantinib for 24 days were fixed in 10% formalin for 30 minutes. After removal of 10% formalin, alizarin red S solution was applied at room temperature in dark for 45 minutes. For von Kossa staining, primary mouse osteoblasts cultured in differentiation medium with or without treating with 100 nM cabozantinib for 24 days were fixed with cold methanol for 20 minutes, followed with the addition of 5% silver nitrate. The culture plates were placed in a UV crosslinker with a setting at 12000 u joules for two cycles.

### Gene array analysis

Duplicate sets of RNAs prepared from PMOs treated with or without cabozantinib for 30 days were subjected to a whole-genome microarray analysis, using the Whole Mouse Genome Oligo Microarray (4x44K, Agilent Technologies) platform (Arraystar Inc., Rockville, MD, USA). Differentially expressed genes between two groups with statistical significance were identified. Array data are deposited in NCBI GEO under accession number GSE90127.

### Ingenuity Pathway Analysis

Ingenuity Pathway Analysis software (IPA, Ingenuity Systems, Inc., Redwood City, CA, USA; http://www.ingenuity.com) was used to identify pathways that are affected by cabozantinib treatment. For network and pathway connection, a dataset with gene identifiers, corresponding fold change and p values was uploaded into IPA with default settings to match the gene types and locations contained in the Ingenuity Knowledge Base. The threshold was set with fold change more than 2 and p value < 0.05 for the IPA program to start core analyses. The canonical pathways involved in upregulated and downregulated genes were sorted based on z-scores provided by IPA program. The Search Diseases and Functions Tool was used to connect the genes encoding extracellular proteins to activities related to “PCa and invasion of tumor cell line”.

### Western blot

Conditioned media were denatured in SDS using 4x SDS sample buffer (Novex) and separated in SDS-PAGE gels (Invitrogen, NuPAGE 4-12% Bis-Tris Gel). The proteins were transferred onto nitrocellulose membrane (Thermo Scientific), immunoblotted with antibodies, and the antibody reactivities detected by ECL (Thermo Scientific). The image intensity was quantified using Image J (NIH Image, Version: 2.0.0-rc-43/1.50e).

### Anchorage-independent growth assay

C4-2B4 cell line, a LNCaP subline generated by serial passages of LNCaP cells in SCID mice [33], was kindly provided by Dr. Robert Sikes (University of Delaware). PC3-mm2 cell line, a PC3 subline generated by serial passage of PC3 cells *in vivo* [34], was kindly provided by I. J. Fidler (M. D. Anderson Cancer Center). C4-2B4 and PC3-mm2 cells were grown at 37°C with 5% CO2 in RPMI medium containing 10% fetal bovine serum. All of the cell lines were routinely verified by short tandem repeat DNA profiling (STR) at M.D. Anderson Cancer Center Characterized Cell Line core. All of the cell lines were routinely checked for mycoplasma contamination using MycoAlert Mycoplasma Detection Kit (Lonza, LT07-218). For soft-agar colony assay, C4-2B4 or PC3-mm2 cells were suspended in 0.35% agarose in RPMI-1640 medium with 5% FBS. The cells were overlaid with top agar contained 0.7% agarose in the same medium. Conditioned medium from PMOs treated with or without cabozantinib was added to the culture medium and the medium was changed every other day. The cells were cultured for 7 days and the cell colonies in each well were counted under microscopy.

### Transwell migration assay

C4-2B4 or PC3-mm2 cells were cultured to confluence in a medium containing 1:1 ratio of RPMI-1640 and α-MEM containing 10% FBS. The cells were then cultured in serum-free medium for 24 hours, trypsinized from the plates, and 0.3 ml cell suspension (30 x 10^4^ cells) was loaded to Transwell chambers. The lower chamber of the migration assay in 24-well plate contained CM (0.5 ml) from osteoblasts treated with or without cabozantinib. The transwell chamber was inserted into the wells and the cells were cultured at 37°C. After incubation for 16 hours, the migrated cells were stained with Calcein AM (Invitrogen). Cells in the upper chamber were removed by using cotton sticks and the cells that migrated through the membrane were quantified in ten randomly chosen fields.

### Statistical analysis

Statistical analyses were performed using Student’s t-test (two-tailed, paired). Data are expressed as the mean ± SD unless otherwise stated. p values less than 0.05 were considered significant.

## SUPPLEMENTARY MATERIALS FIGURE AND TABLE



## Abbreviations

CM: conditioned medium
PCa: prostate cancer
PMOs: primary mouse osteoblasts
real-time RT-PCR: reverse transcription followed with real-time polymerase chain reaction
ELISA: enzyme-linked immunosorbent assay
