# Supplementary material for: Cabozantinib-induced osteoblast secretome promotes survival and migration of metastatic prostate cancer cells in bone
Source: Oncotarget. 2017 Aug 24;8(43):74987–5006. doi: 10.18632/oncotarget.20489 (PMC5650395; doi:10.18632/oncotarget.20489)
Supplement: Supplementary file 1 [file oncotarget-08-74987-s001.pdf]

Cabozantinib-induced osteoblast secretome promotes survival and migration of metastatic prostate cancer cells in bone

SUPPLEMENTARY MATERIALS

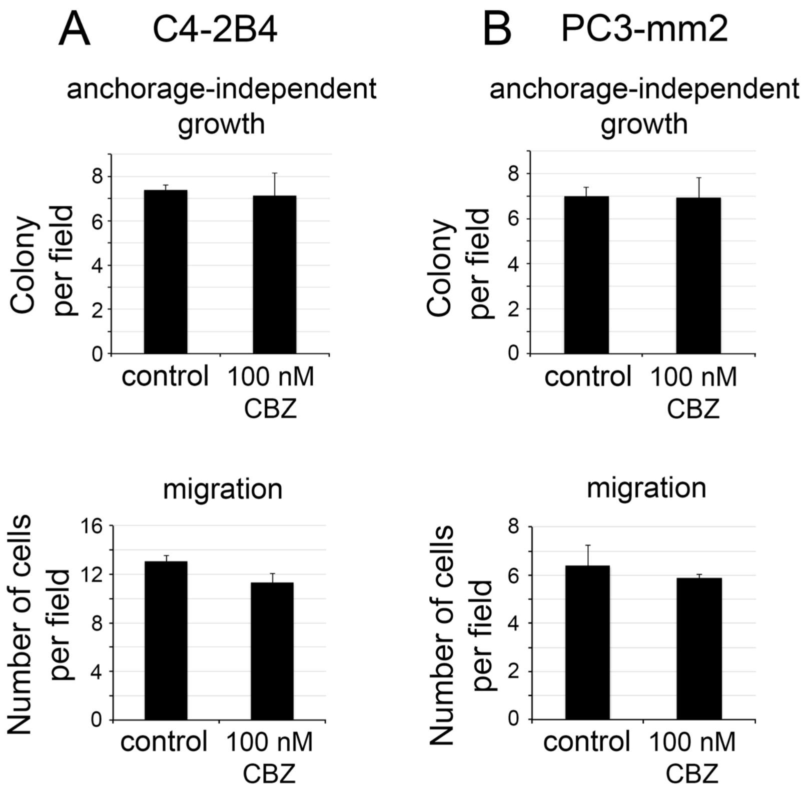

Supplementary Figure 1: Effect of cabozantinib on anchorage-independent growth and migration of C4-2B4 **(A)** or PC3- mm2 **(B)** prostate cancer cells.

Supplementary Table 1: Sequence of primers used in real-time RT-PCR

Gene		Direction	Sequence (5'-3')	Size (base pair)	Product length
TGFβ1	Primer 1	FWD	TGAGT GGCTG TCTTT TGACG	20	293
		REV	TCTCT GTGGA GCTGA AGCAA	20	
DKK1	Primer 3	FWD	GAGGG GAAAT TGAGG AAAGC	20	230
		REV	GGTGC ACACC TGACC TTCTT	20	
WNT-16	Primer 2	FWD	CTAGG CACCA AGGAG ACAGC	20	187
		REV	ACATG CCGTA CTGGA CATCA	20	
LEFTY-1	Primer 1	FWD	TGATT GTCAG CGTGA AGGAG	20	246
		REV	GGAAG CAAAG AGCAC ACACA	20	
PAPPA	Primer 3	FWD	GGCAGTGCTCTGAATCACAA	20	249
		REV	GGGCTCCAAATTAGTGTCCA	20	
IGFBP2	Primer 2	FWD	GGTGTGTGAACCCCAATACC	20	221
		REV	CAAAACTGGGAACTCCTCCA	20	
MET	Primer 3	FWD	GAGAA ACTCT TCGGG CTGTG	20	213
		REV	GTGAG GTGTG CTGTT CGAGA	20	
VEGFR2*	Primer 2	FWD	CAGCT TCCAA GTGGC TAAGG	20	264
		REV	CAGAG CAACA CACCG AAAGA	20	
PLCL1	Primer 2	FWD	ACCAG TTGGG GCAGA TACAG	20	209
		REV	GCCAT TTGCT TGTGG TTTTT	20	
NOTCH2	Primer 3	FWD	GCACG TGTGT TGATG GAATC	20	185
		REV	CTGAC AGTTT TTCCC GGTGT	20	
CAV1	Primer 2	FWD	CCAGC TTCAC CACCT TCACT	20	149
		REV	GCTCT TGATG CACGG TACAA	20	
NFE2	Primer 1	FWD	TGGCC ATGAA GATTC CTTTC	20	123
		REV	GACGG ATGTC CCGAA CTAGA	20	
RANKL	Primer 1	FWD	AGCCG AGACT ACGGC AAGTA	20	208
		REV	GCGCT CGAAA GTACA GGAAC	20	
PTPRV	Primer 1	FWD	ACAGG TGGTC GATGT GTTCA	20	283
		REV	ATAGT CAGGA GAGGG CAGCA	20	
SOX11	Primer 3	FWD	CTGGT GGATA AGGAC CTGGA	20	153
		REV	CGCCT CTCAA TACGT GAACA	20	
ITGA5	Primer 3	FWD	CAAGG TGACA GGACT CAGCA	20	224
		REV	GCTGC AGACT ACGGC TCTCT	20	
DLK1	Primer 1	FWD	CACAC GTTTC GCAAG AAGAA	20	242
		REV	CCACC ACAAA AAGCC AGACT	20	
